# Antibacterial Activity and Cytotoxicity of Immobilized Glucosamine/Chondroitin Sulfate on Polylactic Acid Films

**DOI:** 10.3390/polym11071186

**Published:** 2019-07-15

**Authors:** Ilkay Karakurt, Kadir Ozaltin, Daniela Vesela, Marian Lehocky, Petr Humpolíček, Miran Mozetič

**Affiliations:** 1 Centre of Polymer Systems, University Institute, Tomas Bata University in Zlín, Nam. T.G.M. 5555, 76001 Zlín, Czech Republic; 2 Faculty of Technology, Tomas Bata University in Zlín, Vavreckova 275, 76001 Zlín, Czech Republic; 3 Department of Surface Engineering, Jozef Stefan Institute, Jamova cesta 39, 1000 Ljubljana, Slovenia

**Keywords:** plasma treatment, surface modification, saccharide immobilization, antibacterial activity, count-plate method, cytotoxicity, chondroitin sulfate

## Abstract

Polylactic acid (PLA) is one of the most produced polymeric materials, due to its exceptional chemical and mechanical properties. Some of them, such as biodegradability and biocompatibility, make them attractive for biomedical applications. Conversely, the major drawback of PLA in the biomedical field is their vulnerability to bacterial contamination. This study focuses on the immobilization of saccharides onto the PLA surface by a multistep approach, with the aim of providing antibacterial features and evaluting the synergistic effect of these saccharides. In this approach, after poly (acrylic acid) (PAA) brushes attached non-covalently to the PLA surface via plasma post-irradiation grafting technique, immobilization of glucosamine (GlcN) and chondroitin sulfate (ChS) to the PAA brushes was carried out. To understand the changes in surface properties, such as chemical composition, surface topography and hydrophilicity, the untreated and treated PLA films were analyzed using various characterization techniques (contact angle, scanning electron microscopy, X-ray photoelectron spectroscopy). In vitro cytotoxicity assays were investigated by the methyl tetrazolium test. The antibacterial activity of the PLA samples was tested against Escherichia coli and Staphylococcus aureus bacteria strains. Plasma-treated films immobilized with ChS and GlcN, separately and in combination, demonstrated bactericidal effect against the both bacteria strains and also the results revealed that the combination has no synergistic effect on antibacterial action.

## 1. Introduction

Among all sustainable polymers, polylactic acid (PLA) is one of the most promising and most produced bioplastics due to its superior chemical and mechanical properties, such as biodegradability, good biocompatibility, and low immunogenicity [1,2,3]. These remarkable properties make this aliphatic polyester attractive for medical and biological applications. The usage of PLA, especially in the biomedical field, is increasing constantly and these days PLA is used to produce blood contacting devices, such as implants for bone fixation, screws, pins, sutures, catheters, drug delivery systems, and so on [2,3,4,5]. However, there are a few drawbacks which limit its use in biomedical applications. For instance, due to the presence of pendant methyl groups in structure, PLA is relatively hydrophobic and has low surface energy [1]. The relatively hydrophobic nature of PLA may result in inefficient cell attachment and proliferation, and in some cases, this low cell affinity can lead to inflammation. Another drawback is the lack of reactive functional groups, which reflects in impeding the specific cell interactions and limits PLA’s usage in medical applications [2,3,4,5,6].

The future of PLA utilization in medical applications not only depends on overcoming intrinsic drawbacks, but also on appropriate modification of the surface to achieve new targeted properties or enhance the present features. In this regard, the most important challenges are the activation of the chemically inert PLA surface to reduce hydrophobicity and introduce a new molecules to possess significant physical and mechanical properties, in order to function properly when in contact with blood [7]. Therefore, the material needs appropriate surface treatments without affecting the bulk properties, due to the interactions, which mostly occur at the surface top layer. Some of the commonly used surface treatment methods are gamma irradiation, chemical vapour deposition, ozone treatment, wet chemical methods, UV irradiation, and plasma treatment [8,9,10,11,12]. As compared to other surface treatment methods, plasma technology has obvious advantages, such as non-toxic chemicals, heat-free processing, and modification without changing the bulk properties [13,14,15,16].

In the medical field, especially for implants, microbial colonization and biofilm formation are critical clinical problems, which may lead to long-term treatments, replacement of infected implants, amputations, and even patient death [17,18,19]. In a case when the antibacterial feature is needed, biomedical materials are coated or loaded with various bactericidal compounds. A considerable number of studies have been carried out to provide antibacterial features to polymeric substrates (PLA, PE, PS, PP) with grafting of various antibacterial moieties, such as enzymes [20], saccharides [21], metal ions and nanoparticles [22,23,24], Vitamin E [18], peptides [25,26], quaternary ammonium salts [27].

Amongst these antibacterial moieties, natural-origin saccharides, such as chondroitin sulfate (ChS) and glucosamine (GlcN) are attractive options, since the carbohydrate moieties can imprint both hydrophilic and biocompatibility properties to the material surface [28].

ChS is the most abundant mucopolysaccharide in all of the vertebrates [29]. This naturally occurring glycosaminoglycan (GAG) is an anionic polyelectrolyte. It has garnered much attention in recent years because of its promising properties, such as non-toxicity, biocompatibility, anti-inflammatory activity [30,31], and its potential as an antibacterial and antiviral agent [32]. In addition, the broad spectrum of the biological activities of ChS suggests a potential benefit to the biomedical field, for example, in osteoarthritis, interstitial cystitis and urinary tract infection treatments, cartilage tissue engineering [31], as dietary supplement, in the drug delivery systems [30,33], and in the development of orthopedic materials.

GlcN is a naturally-produced amino sugar, which is essential for glycoproteins, glycolipids, and glycosaminoglycans (GAG). This positively charged saccharide has anti-inflammatory, antioxidant, anti-ageing, anti-cancer, and antibacterial activities [34,35,36,37]. In recent studies, GlcN is observed to be a strong inhibitor to microbial growth, due to its sugar moiety [38,39]. Although the exact mechanism of antibacterial activity of GlcN is still unclear, it is probably caused by the disruption of bacterial cell wall via the free amino group. 

GlcN is also used in biomedical applications in combination with ChS to achieve a synergistic effect [40,41]. For example, the efficacy of GlcN and ChS in treatment of symptomatic osteoarthritis of the knee [42], stimulation of vasculogenesis and angiogenesis [43], and treatment of Kashin-Beck disease [44] have been evaluated in some research. However, fewer researches have been conducted on the combination of GlcN and ChS than on either saccharide alone. In addition, the possible different effects of these saccharides and their combination on the antibacterial activity and cytotoxicity have not been fully clarified. To compare the efficacy of the two saccharides in cell viability and inhibition of the microbial growth more researches need to be done.

Thus, the present study aims to develop a PLA-based antibacterial platform using two common saccharides, which also have cell proliferation and adhesion features, and also to compare the antibacterial and cytotoxic activity of GlcN and CS, individually and in combination. While clinical trials and most in vitro studies have focused on the synergistic effects of glucosamine and CS in treatment of osteoarthritis, the present study focuses on the prevention of bacterial growth on polymer biomaterials by using saccharides. For this purpose, low-pressure radio frequency plasma (RF) method was utilized to activate the PLA surfaces and the molecules of GlcN and ChS were immobilized on the surface of the PLA films to improve in antibacterial activity. The bacterial adhesion of surface modified PLA films was systematically investigated by antibacterial activity against Escherichia coli (E. coli) and Staphylococcus aureus (S. aureus).

## 2. Materials and Methods

Poly(lactic acid) (PLA) 4032 D in pellets form was purchased from Nature Works (Blair, NE, USA). Acrylic acid (99%), sodium metabisulfite (99%), D-glucosamine hydrochloride (99%), chondroitin sulfate A sodium salt (60% balance is chondroitin sulfate C), and sodium hydroxide (98%) were analytical grade reagents and were supplied by Sigma Aldrich (St. Louis, MO, USA).

### 2.1. Preparation of PLA Films

PLA pellets were first dried in a desiccator at 60 °C overnight to eliminate humidity. Then, to obtain PLA sheets, the compression molding technique was used. PLA pellets were hot-pressed at 180 °C for 20min and then the molding plates were placed in another press for cooling. Sheets of PLA about 430 µm thick and 125 × 125 mm square shaped were obtained and then cut into samples of 25 × 25 mm for further surface treatments. The specimens were rinsed with a detergent solution and subsequently dried at room temperature prior to plasma exposure. 

### 2.2. Plasma Treatment

The PLA films were treated using radio frequency (13.56 MHz), low-pressure plasma equipment (model: PICO Diener, Ebhausen, Germany). Briefly, the sample was first inserted into an evacuated to 60 Pa vacuum chamber and 20 sccm of air flowed into the chamber. Each side of the sample was treated by air plasma generated at power 50 W for 60 s.

### 2.3. PAA Grafting and Immobilization of Saccharides

Acrylic acid (AAc) solution was prepared by dissolving 10% of AAc in distilled water with sodium metabisulfite. PLA films were immersed immediately into this homopolymer solution after the irradiation step. Following the grafting reaction, the homopolymer was removed, then the samples were placed into the aqueous solution of 1 w% sodium hydroxide for neutralization.

**Figure 1:**
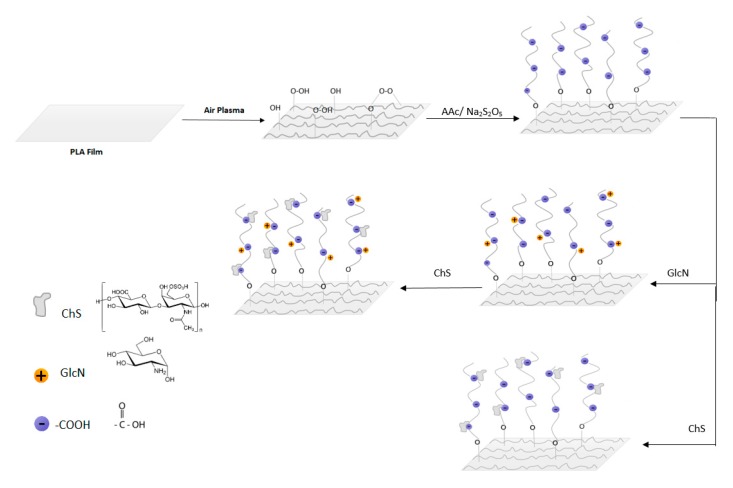
Plasma post-irradiation grafting of Acrylic acid (AAc) onto polylactic acid (PLA) surface followed by immobilization of glucosamine (GlcN) and chondroitin sulfate (ChS) molecules and only ChS immobilization.

For immobilization of antibacterial agents, AAc grafted samples were immersed into either 1 w% GlcN or 1 w% ChS solutions for 24 h. Thereafter, each sample was washed with deionised water. Subsequently, GlcN immobilized samples were lastly placed into 1 w% ChS solution. The films were then removed, washed in distilled water and left for drying overnight for further characterization. All the process is graphically presented in Figure 1.

### 2.4. Characterizations of the Samples

The elemental analysis of untreated and treated PLA films was conducted with X-ray photoelectron spectroscopy (XPS) using the Thermo Scientific K-Alpha XPS system (Thermo Fisher Scientific, Loughborough, UK). A monochromic Al Kα X-ray source (1486.6 eV) line was applied as the photoemission excitation with a 400µm spot size at a power of 72 W.

The change in hydrophobicity induced by surface modifications was analysed through static contact angle measurements by the Surface Energy Evaluation System (SEE System; Advex Instruments, Brno, Czech Republic). As a testing liquid, deionized water was used and digital images of a 2 μL water droplet on the surface were captured by the charged-coupled device (CCD) camera system. Three different spots on the droplet’s image were determined and the contact angle value was obtained for each reading. Each representative contact angle was calculated by averaging at least 10 separate readings for each sample.

The surface morphology of the specimens was observed using a NANOSEM 450 (FEI, Hillsboro, OR, USA) scanning electron microscope at an accelerating voltage of 5.0 kV after sputter coating with gold/palladium.

### 2.5. Cytotoxicity Testing

The proliferation of mouse embryonic fibroblast cells (ATCC CRL-1658^TM^; NIH/3T3) with the disinfected (30 min of exposure to a UV-radiation source) PLA samples were compared by MTT assay. The ATCC-formulated Dulbecco’s Modified Eagle’s Medium (Biosera, Nuaille, France) containing 10% of calf serum and 100 U mL^−1^ penicillin/streptomycin (PAA Laboratories GmbH, Pasching, Austria) was used as the culture medium. The cells were seeded onto square samples (10 × 10 mm) in concentration 1 × 10^5^ cells/mL in volume 200 µL for 1h incubation. The amount of 800 µL of culture medium was added after 1 h incubation and the cells were incubated at 37 ± 1 °C for 72 h. In this study, the cells cultured on polystyrene were used as a reference. 

Cytotoxicity testing was conducted according to the international standard EN ISO 10993-5 with modification. After 72 h incubation, all culture medium was removed and incubated for 4 h with 900µL fresh medium which included 100 µL tetrazolium dye MTT solution (5 mg mL^−1^). Following the removal of all the medium, dimethyl sulfoxide (Merck, Darmstadt, Germany) was added for dissolution of the formed formazan crystals on the sample surfaces. The absorbance was measured at a wavelength of 570 nm (test) and 690 nm (reference) by using an Infinite M200 Pro NanoQuant microplate reader (Tecan, Zürich, Switzerland). Cell viability was shown as the percentage of viable cells after exposure to the tested solutions relative to the reference (100% viability).

### 2.6. Antibacterial Testing

The antibacterial properties of the PLA samples were quantitatively assessed according to an adapted method from the ISO 22196:2007 protocol [45]. As bacteria strains, Staphylococcus aureus (CCM 4516) and Escherichia coli (CCM 4517) were used.

Briefly, PLA films were sterilized by immersion in 70% ethanol for 24 h. Test bacteria were transferred to nutrient broth (NB) and for using as test inoculations, the number of bacteria was adjusted to certain concentrations, which is between 2.5 × 10^5^ cells/mL and 1 × 10^6^ cells/mL, with dilution. Both treated and untreated samples (25 mm × 25 mm) were placed, in groups of tree, into Petri dishes and 0.4 mL of the test inoculum was pipetted onto each sample, which was then covered with a polypropylene (PP) film (20 × 20 mm). The Petri dishes were incubated for 24 h at 35 °C. After the incubation, both the samples and PP covers were washed with 10 mL of neutralizing broth. The recovered bacterial suspensions were diluted to 10-fold concentrations. Each diluted and undiluted bacterial solution was placed into Petri dishes with 15 mL of count plate agar. The number of bacteria colonies were counted after 24 h of incubation.

## 3. Results and Discussion

### 3.1. Surface Chemistry and Morphology

**Figure 2:**
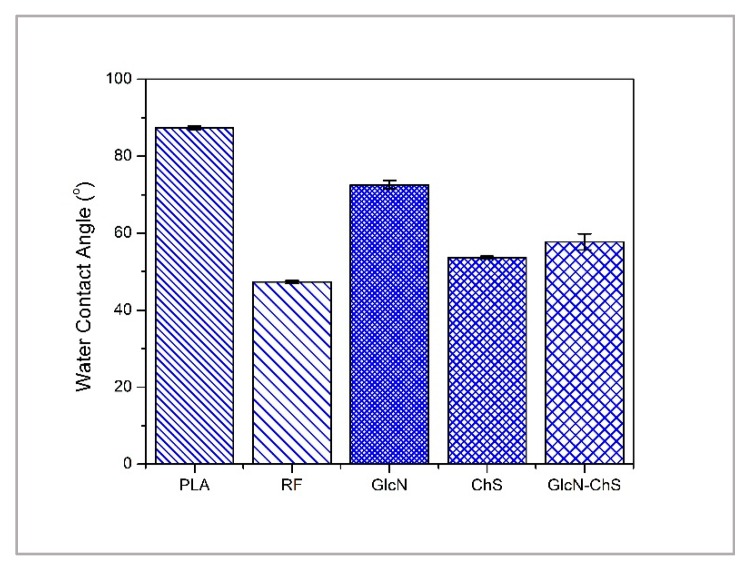
Water contact angles of untreated and treated PLA. Results represent mean value from three independent experiments with standard deviations.

Through static contact angle measurements, surface modification, which induced a change in hydrophilicity, was analyzed (Figure 2). As can be seen, the static water contact angle of untreated PLA was 87°. This makes it highly hydrophobic and difficult for further surface treatment because of lacking polar and functional groups on the PLA surface. The value of the contact angle decreased significantly to 47° after plasma treatment as a result of the presence of plasma-induced hydrophilic oxidative functional groups and surface roughening. The GlcN immobilization to PLA resulted in an increased hydrophobicity but still remained lower than untreated PLA. In contrast, PLA was modified by the ChS and led to a lower contact angle value, which can be connected to the more hydrophilic character derived from hydroxyl, carbonyl and amine groups of ChS. The immobilization of ChS after GlcN decreased the hydrophobicity of only GlcN coated sample. The results preliminarily suggest successful modification of the polymer surfaces, which is supported by the difference between the morphology of untreated and treated PLA surfaces.

**Figure 3:**
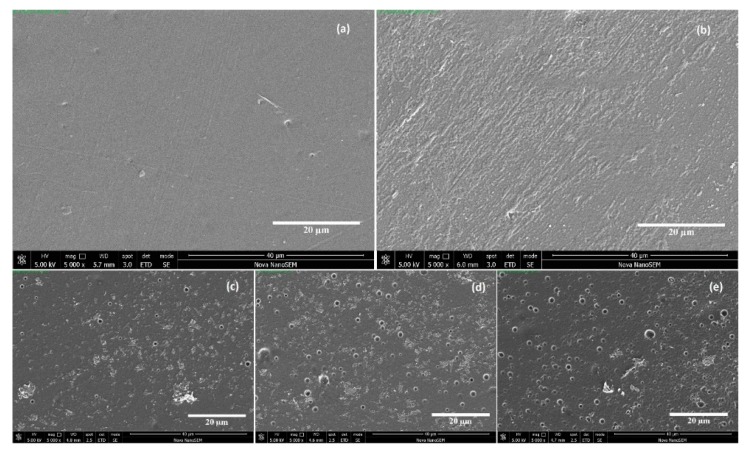
SEM images of the (**a**) untreated PLA; (**b**) plasma treated PLA; (**c**) ChS; (**d**) GlcN; (**e**) GlcN-ChS grafted films.

Typical SEM micrographs of the pristine and modified PLA films are illustrated in Figure 3. The scale bar represents 20 μm. It can be seen that the surface of the untreated PLA sample is homogenous, and relatively smooth at a micrometric scale. Due to the compression molding technique, a minor fiber-like features are observed. After plasma treatment, the PLA surface possesses a rougher appearance, as a consequence of the surface functionalization and etching processes. This increased roughness is the desired surface condition for further immobilization steps. When the results are evaluated together with the contact angle values given in Figure 2, it can be said that plasma treatment leads to an improvement in hydrophilicity. Figure 3c–e present the images of the surfaces on which saccharide aggregates are clearly seen. ChS and GlcN were deposited as heterogeneous bidimensional layers on PLA surfaces. 

**Figure 4:**
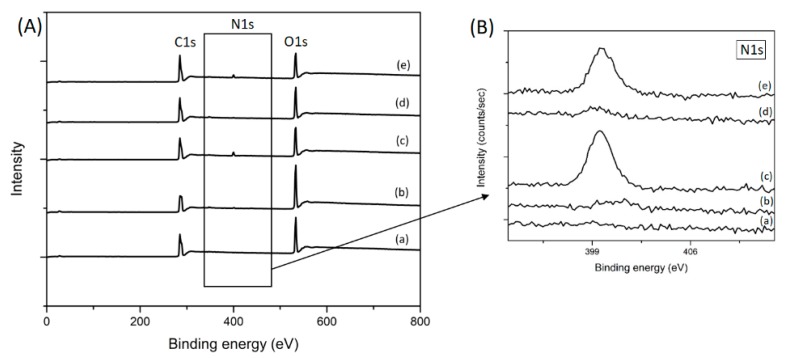
High resolution XPS spectra of (**A**) PLA films (a) untreated PLA; (b) plasma treated PLA; (c) ChS; (d) GlcN; (e) GlcN-ChS grafted films. (**B**) N1 core level spectra of PLA.

For the analysis of surface chemical compositions of the PLA surfaces, XPS measurements were carried out before and after the treatments. Figure 4 shows the XPS full spectra of the samples with their corresponding surface elemental compositions and N1 core level spectra. The XPS spectra (Figure 4A) for all samples show two main contributions corresponding to C(1s) at 285 eV and O(1s) located at 533 eV, due to the chemical structure of PLA. After air-plasma treatment, an increase in the peak intensity corresponding to the O(1s) transition can be clearly seen, due to the presence of oxide functional groups. In addition, a small peak that corresponds to the contribution of nitrogen, N(1s), with a binding energy of 400 eV is observed in the plasma treated sample. This nitrogen content stems from the air plasma application, which is mainly composed of oxygen (O) and nitrogen (N) radicals. After GlcN immobilization, it is expected that the nitrogen element appears on the PLA film surface. However, from the XPS spectra of GlcN immobilized PLA films (Figure 4A-d), no obvious N1s peak can be detected. This might result from the low amount of GlcN immobilized on the PLA surface. Figure 4B-c,B-e show the increase in the intensity of the nitrogen peak, which indicates ChS presence on the PLA surface. 

The elemental content of carbon, oxygen, nitrogen, and sulfur in each sample is summarized in Table 1. According to the chemical structure of PLA, the XPS analysis of the untreated films indicates that the surface is dominated by carbon (67.8%) and oxygen (31.9%) species. The increased O/C ratio in the RF samples indicates the presence of oxide functional groups just after the air plasma treatment. As shown in the table, untreated PLA films had no nitrogen and sulfur elements, whereas the nitrogen contents were observed for all the other samples, and sulfur contents were found for ChS and GlcN-ChS immobilized samples with the levels of 0.1% and 0.3%, respectively. These nitrogen and sulfur contents are the proof for successful activation of the PLA surface with plasma treatment and grafting of the saccharides.

### 3.2. Cytotoxicity of PLA Films

Cell viability was quantified by an MTT assay as an indicator of mitochondrial succinate dehydrogenase enzyme activity. The viability of the reference corresponds to 100% survival of cells in the absence of the tested substances in the cultivation medium. According to the EN ISO 10993-5, values above 80% compared to reference are assigned to ‘no cytotoxicity’, values from 60% to 80% ‘mild cytotoxicity’, and values below 40% ‘severe cytotoxicity’ [46].

**Figure 5:**
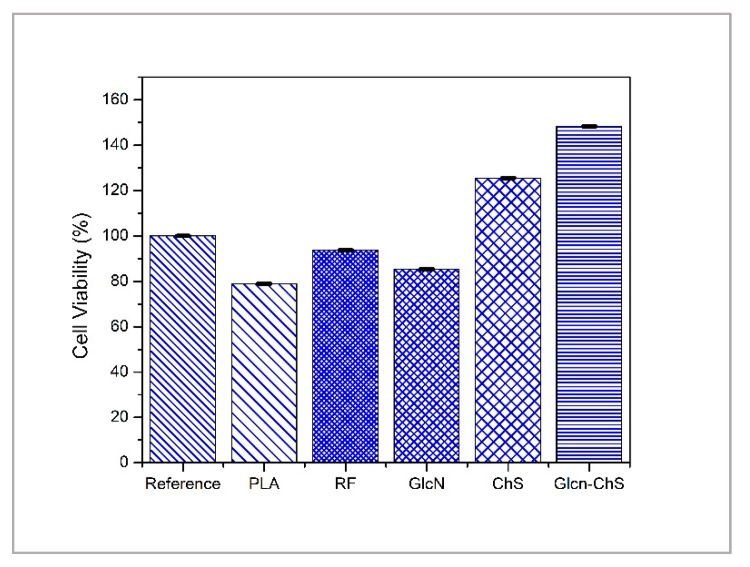
Cytotoxicity of untreated-treated PLA and polystyrene as reference. Error bars represent the standard deviation of the three independent experiments.

Cytotoxicity data are presented in Figure 5. All of the results obtained from the cytotoxicity assay resulted in significant readings as indicated by *p* < 0.05. It can be seen that all of the relative cytotoxicity values (%)-except for untreated PLA-are higher than 80%, independent of polymer modification. One possible reason for this phenomenon is the increased hydrophilicity of untreated PLA with various modifications (plasma treatment, grafting). The GlcN grafted samples display 85% cell viability, while the ChS immobilized samples have more than 120% viability of the cells. For the combination of these two saccharides, the cell viability increases to 148%, which shows better biocompatibility than either saccharide alone.

Other studies reported the evaluation of toxicity of chondroitin sulfate and glucosamine. Most of the studies claim little or no cytotoxicity of either GlcN or ChS for concentrations below 5.0 mg/mL [47,48]. One study reported the combination of GlcN and ChS resulted in an increase in cellular metabolic activity in chondrocytes monolayer cultures (cell viability 139%) upon 7 days of incubation, which is consistent with our findings [49]. In another study, an enhancement in cell proliferation was found with these two saccharides [50].

### 3.3. Antibacterial Performance of PLA Films

The antibacterial activity of the PLA films was evaluated against *Staphylococcus aureus* and *Escherichia coli* strains, and analyzed by comparing the number of viable cells in the agar plates after 24h of incubation time. As shown in Table 2, untreated PLA has antibacterial activity against neither bacteria strains. After plasma treatment (RF) a similar number of viable bacteria of untreated PLA is found, which indicates the absence of any bactericidal effect before surface coating with suitable agents. While the best antibacterial activity is observed with only ChS immobilized samples against E. coli bacteria strains (<1 means no colonies recovered), the highest antibacterial activity against the S. aureus strains is exerted by only GlcN attached PLA films with 1.9 cfu/cm^2^. The difference in counts between GlcN combined with ChS immobilized samples and separately attached saccharides are small and this combination results in a destruction of more than 99.99% of the inoculation, which generally is accepted as the definition of bactericidal agents [51,52]. 

## 4. Conclusions

The antibacterial surface modification of PLA films was achieved through the immobilization of GlcN and ChS on film surfaces via plasma treatment technique, followed by AAc grafting. The contact angle and XPS results verify successful immobilization of the saccharides. In the survey scan XPS spectra increases in characteristic elements (N and S) of ChS and GlcN were observed. SEM images showed that the saccharide aggregates partially covered the PLA film surfaces. The antibacterial testing results demonstrated that PLA films coated with ChS exhibited the highest antimicrobial activity against E. coli. Besides, only-GlcN immobilized PLA films showed the best bactericidal effect against S.aureus. When combined with ChS the degree of both bacteria growth inhibition was still up to 99.99%.

This study definitely proved that the developed GlcN/ChS coated PLA films are excellent bactericide agents against representative gram-positive and gram-negative bacteria. Furthermore, the combination of these two saccharides should be highlighted in the current study, due to increased cell viability, which could make it easier to bring the developed medical devices to the market. 

## Figures and Tables

**Table 1:** The atomic weight percentage of unmodified and modified polylactic acid (PLA) samples.

Sample Type	Composition (%)				Ratio
	C	O	N	S	O/C
PLA	67.8	31.9	-	-	0.47
RF	60.6	38.2	0.9	-	0.63
GlcN	69.4	29.6	0.5	-	0.44
ChS	66.3	28.9	4.4	0.1	0.43
GlcN-ChS	70.7	25.1	3.6	0.3	0.36

**Table 2:** The number of viable bacteria on the PLA films.

	Sample	Initial CFU	PLA	RF	GlcN	ChS	GlcN-ChS
Bacteria							
S.aureus *CCM 4516* N (cfu/cm^2^)		2.0 × 10^6^	1.8 × 10^5^	2.1 × 10^5^	1.9	8.8	1.1 × 10^1^
*E.coli* *CCM 4517* N (cfu/cm^2^)		2.2 × 10^7^	2.3 × 10^6^	2.2 × 10^6^	7.8	<1	7.7 × 10^1^
